# Difficulties and flaws in performing accurate determinations of zeta potentials of metal nanoparticles in complex solutions—Four case studies

**DOI:** 10.1371/journal.pone.0181735

**Published:** 2017-07-27

**Authors:** Sara Skoglund, Jonas Hedberg, Elena Yunda, Anna Godymchuk, Eva Blomberg, Inger Odnevall Wallinder

**Affiliations:** 1 KTH Royal Institute of Technology, Division of Surface and Corrosion Science, School of Chemical Science and Engineering, Stockholm, Sweden; 2 National Research Tomsk Polytechnic University, Tomsk, Russia; 3 National University of Science and Technology “MISIS”, Moscow, Russia; 4 RISE Research Institutes of Sweden, Chemistry, Materials and Surfaces, Borås, Sweden; Institute of Materials Science, GERMANY

## Abstract

The zeta potential (ZP) is a parameter commonly used to characterize metal nanoparticles (NPs) in solution. Such determinations are for example performed in nanotoxicology since the ZP influences *e*.*g*. the interaction between cells and different biomolecules. Four case studies on different metal NPs (Cu and Zn NPs, and citrate capped Ag NPs) are presented in this study in order to provide guidance on how to accurately interpret and report ZP data. Solutions of high ionic strength (150 mM NaCl) induce a higher extent of particle agglomeration (elucidated with Ag NPs) when compared with conditions in 10 mM NaCl, which further complicates the prediction of the ZP due to *e*.*g*. sedimentation and broadening of the zeta potential distribution. The particle size is seldom included specifically in the standard ways of determining ZP (Hückel and Smoluchowski approximations). However corrections are possible when considering approximations of the Henry function. This was seen to improve the analysis of NPs, since there are cases when both the Hückel and the Smulochowski approximations are invalid. In biomolecule-containing cell media (BEGM), the signal from *e*.*g*. proteins may interfere with the measured ZP of the NPs. The intensity distribution of the ZP of both the blank solution and the solution containing NPs should hence be presented in addition to the mean value. Due to an increased ionic strength for dissolving of metal NPs (exemplified by Zn NPs), the released metal ions must be considered when interpreting the zeta potential measurements. In this work the effect was however negligible, as the particle size was several hundred nm, conditions that made the Smoluchowski approximation valid despite an increased ionic strength. However, at low ionic strengths (mM range) and small-sized NPs (tens of nm), the effect of released metal ions can influence the choice of model for determining the zeta potential.

Sonication of particle dispersions influences not only the extent of metal release but also the outermost surface oxide composition, which often results in an increased ZP. Surface compositional changes were illustrated for sonicated and non-sonicated Cu NPs. In all, it can be concluded that accurate measurements and interpretations are possible in most cases by collecting and reporting complementary data on characteristics such as particle size, ZP distributions, blank sample information, and particle oxide composition.

## Introduction

The use of nanoparticles (NPs) is constantly increasing [1–4]. With this increased usage comes the challenge to identify and assess potential risks that these particles might induce on humans and the environment. When performing toxicological studies on NPs it is important to thoroughly characterize their physicochemical properties, both as pristine particles and after interactions with the biological/environmental media of interest. Such a characterization enables an improved understanding of the underlying toxicological mechanisms [5, 6], and assists in the prediction of their effects on human health and on the environment.

The zeta potential (ZP) is the apparent surface potential that is related to the surface charge. The ZP is an important parameter to assess when studying NPs in suspension as it for instance affects particle agglomeration, sedimentation, interaction and complexation with other media constituents [7]. The mentioned parameters are all known to influence the biological effect of NPs [6, 8, 9]. Surface-functionalized cationic NPs are in general more cytotoxic than neutral or anionic NPs, *e*.*g*. because they are more prone to induce lysosomal damage [10–16]. Positively charged NPs were in a study by Bhattacharjee *et al*.[10] shown to be more cytotoxic compared with negatively charged and neutral particles. The authors attributed this effect to an increased induction of oxidative stress. Moreover, McGuinnes *et al*.[11] showed that the extent of interaction between particles and cell surfaces or organelle membranes is affected by the ZP of the particles. Cho *et al*. showed that metal-containing NPs with high absolute ZPs could induce lung inflammation [12]. Thus, the ZP of the NPs can affect cellular damage and uptake. The NP uptake by the cells is an important parameter that has been discussed in many papers [17–21].

It is not trivial to determine the ZP of NPs in *e*.*g*. complex biological environments [13]. The surface charge of the particles (and thus the ZP) is known to be influenced by several properties of the dispersion media and by pH in particular [5, 14]. Biological fluids contain proteins and other macromolecules that adsorb to different extent to the NPs, often denoted as the bio-corona. The characteristics of the bio-corona continuously changes, in particular as the NPs move between different chemical surroundings [15]. The presence and nature of adsorbed molecules will change the overall charge of the NPs and hence their ZP.[1, 16, 17] Different theoretical models that can be used for ZP calculations of bare and polymer coated NPs have been proposed by Doane *et al*.[18] Other challenges to enable accurate assessment of the ZP of NPs are their small size, polydisperse systems, non-spherical particles, and unstable suspensions in terms of sedimentation and dissolution (in the case of metal NPs) [18–20]. In an interlaborative comparative study, a good repeatability of size measurements on monodisperse NPs suspensions was shown, whereas it demonstrated the need of improved methods and more detailed protocols to increase the repeatability for polydisperse suspensions [20]. The same conclusions were drawn for the ZP measurements [20]. The need of improved and detailed protocols for NP characterization has also been addressed elsewhere [10, 21–23].

The aim of this study is to give advice on how to interpret and report measurements on ZPs of metal NPs in complex solutions of relevance for studies on nanotoxicology and environmental interactions. Potential pitfalls and misunderstandings of ZP measurements and interpretations are elucidated in four case studies, all revealing common difficulties and flaws that may be encountered when performing ZP measurements of metal NPs. The case studies aim at elucidating the effects of often pronounced agglomeration of metal NPs in media of high ionic strength, the interference of biomolecules, the effect of rapid release of metal ions and complexes, and the influence of probe sonication on the ZP. To this end, ZP and size distribution measurements have been conducted on 10 nm citrate coated silver Ag NPs in NaCl solutions of both low and high ionic strengths (10 mM and 150 mM NaCl, respectively), as well as in a cell medium, Bronchial Epithelial Cell Growth Medium (BEGM). The effect of dissolution was addressed by performing measurements of ZP, size distribution and metal release on rapidly dissolving Zn NPs. The effect of probe sonication on the ZP was illustrated with sonicated and non-sonicated Cu NPs in ultrapure water.

## Materials and methods

### Nanoparticles

#### Ag NPs

10 nm Citrate BioPure^™^ Ag NPs were purchased (1 mgmL^-1^ stock dispersions, in 2 mM citrate) from NanoComposix Inc. (San Diego, CA, USA). The behavior of the Ag NPs in cell medium (BEGM, Lonza, Sweden) in terms of agglomeration and release of silver as well as their toxicological profile have been thoroughly examined in a previous study by some of the authors [24].

#### Zn NPs

The Zn NPs were supplied by LLC Advanced Powder Technologies, Tomsk, Russia. They were obtained by electrical explosion of zinc wires in gaseous argon atmosphere with 10 vol. % hydrogen at an excess pressure of 0.15 MPa and a charge capacitance of a capacitive accumulator (24 kV). The diameter and length of the zinc wires were 0.3 and 75 mm, respectively.

#### Cu NPs

The Cu NPs were produced by means of wire explosion, similar to Zn NPs by the same producers, and have been characterized in detail elsewhere [25–27].

### Solutions and dispersion preparation

10 and 150 mM NaCl (purchased form Sigma Aldrich, reagent grade) solutions were prepared by mixing the salt in ultrapure water (18.2 MΩ cm, Millipore, Sweden). The cell medium BEGM (Lonza, Sweden) solution was used as received and stored under cold and dark conditions. The 1 mg mL^-1^ stock solution of citrate coated Ag NPs was diluted without further treatment to 10 mgL^-1^ prior to the ZP and size distribution measurements. This particle concentration and sample preparation are relevant for toxicological studies.[24]

The Cu NPs were dispersed using a sonication probe for 15 min in ultrapure water, resulting in 7056 J of delivered acoustic energy, see details elsewhere [28]. For the measurements of XPS after sonication, the dispersed Cu NPs were centrifuged (3000 rpm, Eppendorf 5702 centrifuge, USA) for 15 min to collect the NPs for analysis.

Zn NPs were dispersed at concentrations of 10 mgL^-1^ and 100 mgL^-1^ in artificial surface water (OECD 203 medium, ISO 6341). The surface water was prepared using ultrapure water (18.2 MΩ cm, Millipore, Sweden) and analytical grade chemicals, according to Table 1. The solution pH was adjusted with a 0.95% solution of H_2_SO_4_. The samples were sonicated (30% duty cycle, output control 5, Branson Sonifier 250, Emerson, USA) for 2 min prior to exposure.

**Table 1:** Chemical composition and pH of OECD surface water.

**Chemical reagent, mgL**^**-1**^	**CaCl**_**2**_**·2H**_**2**_**O**	**MgSO**_**4**_**·7H**_**2**_**O**	**NaHCO**_**3**_	**KCl**
	29.4	12.3	6.48	0.58
**pH**	6.0			
**pH adjustment**	≈170 μL 0.95% H_2_SO_4_			

### Zeta potential

Zeta potential (ZP) measurements were carried out using a Malvern Zetasizer Nano ZS instrument (Malvern Instruments, UK). Triplicate samples were measured three times each at 21°C. The measurements were initiated within 3 min after sample preparation.

### Size distributions

Size distribution and agglomeration behavior of particles in suspension were evaluated by means of dynamic light scattering employing photon cross correlation spectroscopy (PCCS) (NanoPhox, Sympatec GmbH, Germany) at 21±2°C. The measurements were initiated within 3 min after sample preparation. Triplicate samples were investigated and data from the unique measurements was integrated to produce a single distribution with the PCCS software (Windox 5). Standard latex samples (20 ± 2 nm) (Sympatec GmbH, Germany) and blank samples were analyzed prior to the measurements to ensure a high accuracy of the measurements.

The volume distribution was determined for the Zn and Ag NPs, which required the input of the refractive index for these materials (for 632.8 nm wavelength used in the PCCS); 0.118 + 5.72 i for Zn and 0.13 + 3.99 i for Ag.

All analysis assumed spherical NP agglomerates, an assumption that may not always comply with reality. The interpretations of particle sizes should hence not be too specific, but rather rely on relative comparisons and trends.

### Metal release

The extent of released zinc from the Zn NPs into artificial surface water was determined by exposing the suspensions and corresponding blank solutions (only OECD 203 test media, no added Zn NPs) for 0.25, 0.5 and 1 h at 21°C in closed vessels under slight agitation (bi-linear, 25 rpm). After the predetermined exposure times, the supernatants were removed using a pipette dispenser and consecutively centrifuged for 10 min (3000 rpm, Eppendorf 5702 centrifuge, USA). Successful removal of the particles from the solution was confirmed by PCCS, as defined when the scattered light intensities were equal to the background level. This background level corresponds to a detection limit of NPs of approx. 30 μgL^-1^, as deduced by Ag NPs in a previous study [29]. After removal of the NPs, all solutions were pH adjusted to a pH<2 using 30 μL of ultrapure 65% HNO_3_ for preservation and avoidance of metal complexation. Metal release analyses were carried out by means of flame atomic absorption spectroscopy (F-AAS, Perkin Elmer AAnalyst 800, USA). All measured concentrations were based on triplicate readings for each sample. Calibration of the instrument was made with calibration standards 0, 1, 3 and 10 mgL^-1^. The limit of detection (LOD) for Zn was 20 μgL^-1^ and the limit of quantification (LOQ) was 50 μgL^-1^. All reported values are above the LOQ and presented with corresponding blanks subtracted. The recovery for all samples exceeded 95%.

### XPS analysis

X-ray photoelectron spectroscopy analyses were performed using a Kratos AXIS UltraDLD X-ray photoelectron spectrometer (Kratos Analytical, UK) using a monochromatic Al X-ray source (150 W). Sonicated NPs were applied on copper tape (no contribution to the generated Cu 2p signal), dried under a stream of nitrogen, and stored in a desiccator for approx. 1 h before insertion in the XPS chamber for analysis. The non-sonicated Cu NPs were immersed in ultrapure water for the same duration as the sonication procedure (15 min) and applied on the copper tape as described above. Measurements were performed on two separate areas for each sample (approx. sized 700 x 300 μm). Detailed spectra with a pass energy of 20 eV were acquired for Cu2p, O1s and C1s. All binding energies were adjusted to the C1s contamination peak (C-C, C-H) at 285.0 eV.

### Transmission electron microscopy

Transmission electron microscopy (TEM) images for Cu and Zn NPs were acquired using a Hitachi HT7700 instrument. The NPs were dispersed on the copper grids with holey fibers (Ted Pella, USA) by first sonicating 1 gL^-1^ NPs in butyl alcohol, followed by pipetting of a drop of solution that was left to evaporate overnight, before analysis. The sonication routine was the same as described above. The TEM images were collected in bright field mode.

A different equipment and preparation method was used for the Ag NPs. A Tecnai 10 instrument (Fei, Netherlands) was used, with an acceleration voltage of 100 kV, The camera employed was a Mega View III digital camera (Soft Imaging System, GmbH, Munchen, Germany). The grids were prepared by dropping the particle dispersion (approx. 3 μL) onto the grids, followed by 5 min in air, and subsequent removal of the liquid using filter paper.

### Theory

The electrical double-layer surrounding a charged particle results from counter ions (ions of opposite charge) in the media that accumulate close to the particle surface where they become strongly bound to the particle, making up the inner layer (the Stern layer). Ions of the same charge (co-ions) as the particle surface are depleted close to the surface and increases with the distance from the surface. The counter ions decreases in concentration with the distance from the surface, following the Boltzmann distribution, and together with the co-ions, creating a region with more loosely associated ions (the diffuse layer). The ion cloud of the two layers makes up the electrical double layer [30]. The Debye length (screening length) is a measure of the thickness of the electrical double layer (*i*.*e*. how far it extends from the surface). *κ* [m^-1^] is the inverse of the Debye screening length, expressed by Eq 1.

$$\kappa^{-1}={\left[\frac{\varepsilon_{r}\varepsilon_{0}k_{B}T}{2N_{A}e^{2}I}\right]}^{1/2}$$ (1)

with *ϵ*_*o*_ [8.854·10^−12^ C^2^ J^-1^ m^-1^] being the permittivity of vacuum *ϵ*_*r*_ the unitless dielectric constant of the solution, *N*_*A*_ [6.022·10^23^ mol^-1^] Avogadro’s constant and *I* [moldm^-3^] the ionic strength. The charge difference causes a potential that changes depending on the distance from the surface. Further discussions and details regarding this double-layer definition are given elsewhere [30, 31]. When the particle moves in the solution, ions out to a certain distance (generally somewhere in the diffuse layer) from the particles surface will move with it. This distance is called the slipping plane or hydrodynamic shear plane, and it is at this distance from the particle surface that the ZP is measured, i.e. at some distance from the particle surface.

When particles are positioned in an external electrical field, they will move with a certain speed and direction depending on the potential of the electric field and the size of the particles [30]. The most common way to measure the velocity of the particles is called Phase Analysis Light Scattering, from which the electrophoretic mobility (*U*_*E*_) is calculated. When estimating the ZP from the measured electrophoretic mobility, a number of assumptions have to be made depending on the method used for the conversion. The Henry equation (Eq 2) is often used for the conversion:

$$U_{E}=\frac{2*\varepsilon *ZP*f\left(\kappa *a\right)}{3*\eta }$$ (2)

where ε is the dielectric constant, ZP [mV] the zeta potential and η the viscosity [kgs^-1^m^-1^]. Eq 1 also contains the Henry function f(κ·a), where κ is the inverse screening length of electrostatics [m^-1^] and *a* is the particle radius [nm] [32]. With the Henry equation follows a number of possible approximations, usually either in line with the approaches of Smoluchowski or Hückel, according to which f(κ·a) is assumed to be 1 (Hückel) or 1.5 (Smoluchowski) [7]. The main difference between the two approximations is that the Smoluchowski approximation assumes that the electrical double layer thickness is much thinner than the particles themselves [19, 33, 34], while the Hückel approximation instead assumes the double layer to be much thicker than the radius of the particles [7, 18, 30]. An expression that bridges these two values by taking the particle size into account has been proposed by Ohshima *et al*.[7, 35], expressed by Eqs 3 and 4:

$$f\left(\kappa *a\right)=1+\frac{1}{{\left(1+\delta \right)}^{3}}$$ (3)

$$\delta =\frac{5}{2\kappa *a\left(1+2e^{-\kappa *a}\right)}$$ (4)

Eq 3 is an approximation for the Henry function and converges to the Hückel approximation for small NPs and low ionic strengths, and to the Smoluchowski approximation for large sized particles and relatively higher ionic strengths [36]. The Ohshima correction is a way to derive values for conditions when both the Hückel and the Smoluchowski approximation are invalid.

In this paper we have employed the Smoluchowski approximation in the figures of ZP distributions if not stated differently, just for illustration.

## Results and discussion

### NP characterization

**Fig 1:**
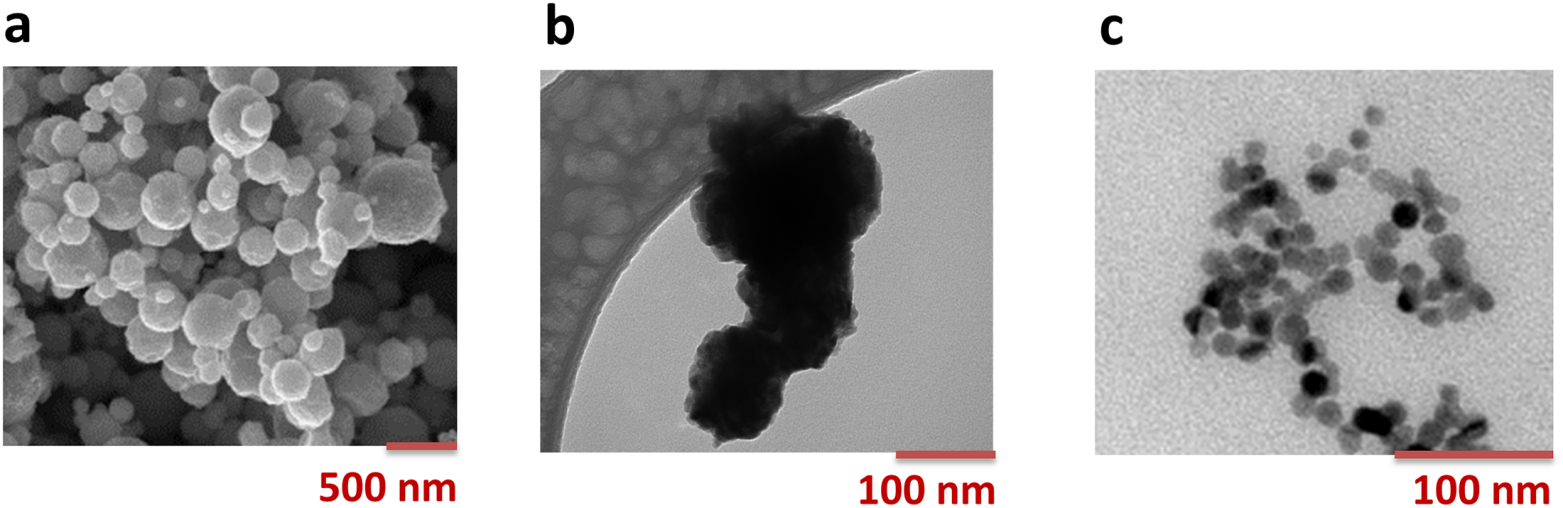
TEM images of the studied NPs. a: Zn NPs, b: Cu NPs, c: Ag NPs.

TEM images of the studied Zn, Cu and Ag NPs are shown in Fig 1. The Zn NPs were mainly spherical, while the Cu NPs were close to spherically shaped, but slightly more irregular. Additional TEM images of the Ag NPs are available elsewhere, showing spherically shaped 10 nm primary particles [37]. Citrate was used as a capping agent for the Ag NPs. XPS investigations revealed the surface oxide of the Cu NPs to comprise both CuO and Cu_2_O [38]. The BET areas of the NPs were 13.6 and 7.23 m^2^/g for the Zn and Cu NPs, respectively.

### Case study 1: Particle agglomeration is largely governed by the ionic strength of the solution

**Fig 2:**
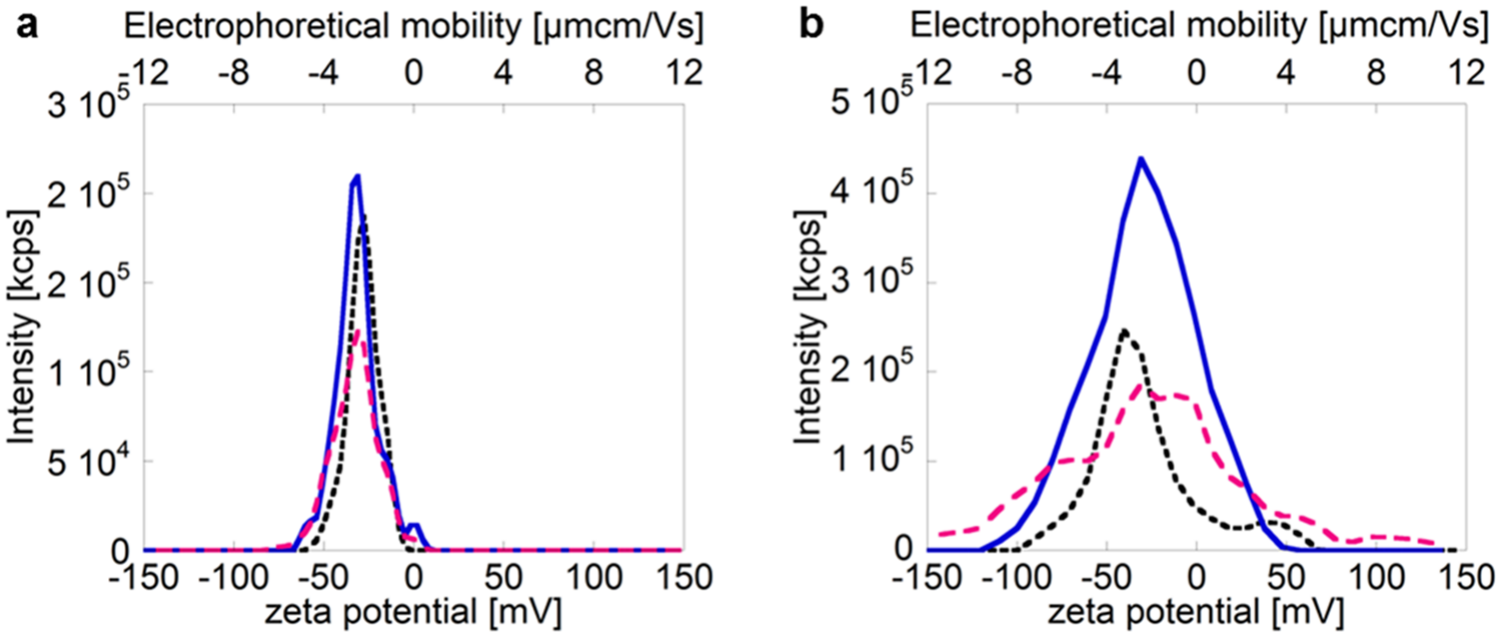
Zeta potential intensity distributions for three separate measurements of 10 nm citrate coated AgNPs (particle concentration: 10 μg mL^-1^) in (a) 10 mM NaCl and (b) 150 mM NaCl, with intensity (kcounts s^-1^) on the y-axis and zeta potential (mV) plus electrophoretic mobility (μmcmV^-1^s^-1^) on the x-axes. The different curves represent three consecutive measurements of the same sample. The zeta potentials were determined using the Smoluschowski approximation.

The first case study comprises of a comparison between citrate-capped Ag NPs dispersed in 10 mM NaCl and 150 mM NaCl. As previously emphasized, it is essential to carefully evaluate and report the intensity distribution of the ZP as a complement to the mean value. The appearance of the intensity distribution curve can vary significantly depending on the solution composition and sample preparation. This is elucidated in Fig 2, which shows ZPs and electrophoretic mobilities of Ag NPs determined in 10 mM and 150 mM NaCl, respectively.

Considerations of only the peak zeta potentials only in the distributions yield significant differences between the Ag NPs in 10 and 150 mM NaCl (p<0.05, Student’s t-test). However, the curves for 150 mM NaCl are broader and the reproducibility is worse compared with the measurements in 10 mM NaCl.

**Fig 3:**
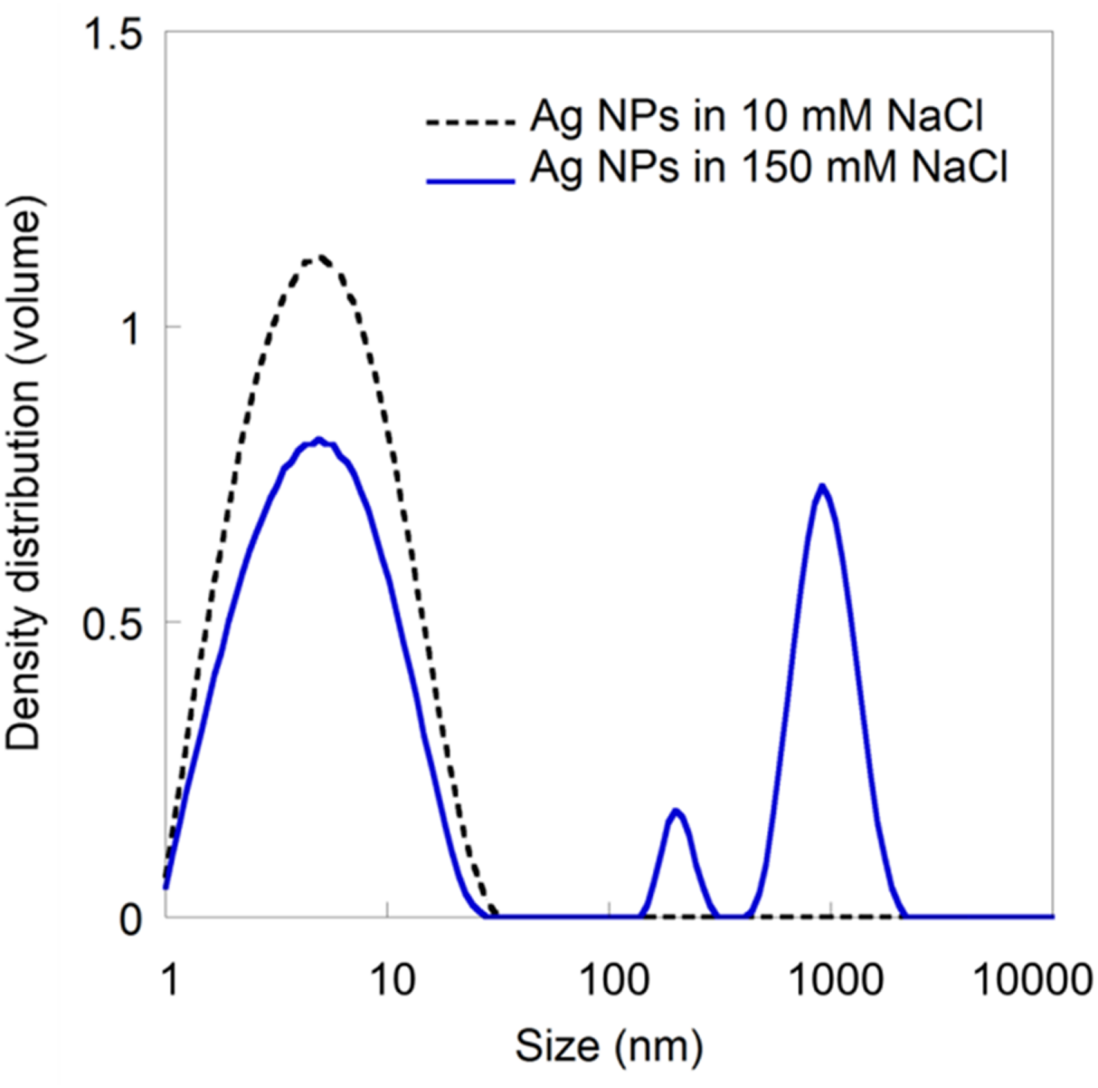
Particle size distribution measurements (volume) by means of PCCS of citrate-capped Ag NPs in 10 mM and 150 mM NaCl, respectively.

This broadening of the zeta potential distribution is, in different ways, connected to the higher ionic strength. The increased ionic strength results in an enhanced screening of surface charges and reduction of the Debye length (Eq 1) [39], which induce agglomeration for the 150 mM NaCl solution, as seen in Fig 3. This agglomeration (particle sizes >100 nm) was statistically significant when comparing the two concentrations (10 and 150 mM NaCl, p<0.05, Student’s t-test). A reduction in thickness of the electric double layer with increased ionic strength will furthermore result in a reduced ZP [32, 40–42].

**Fig 4:**
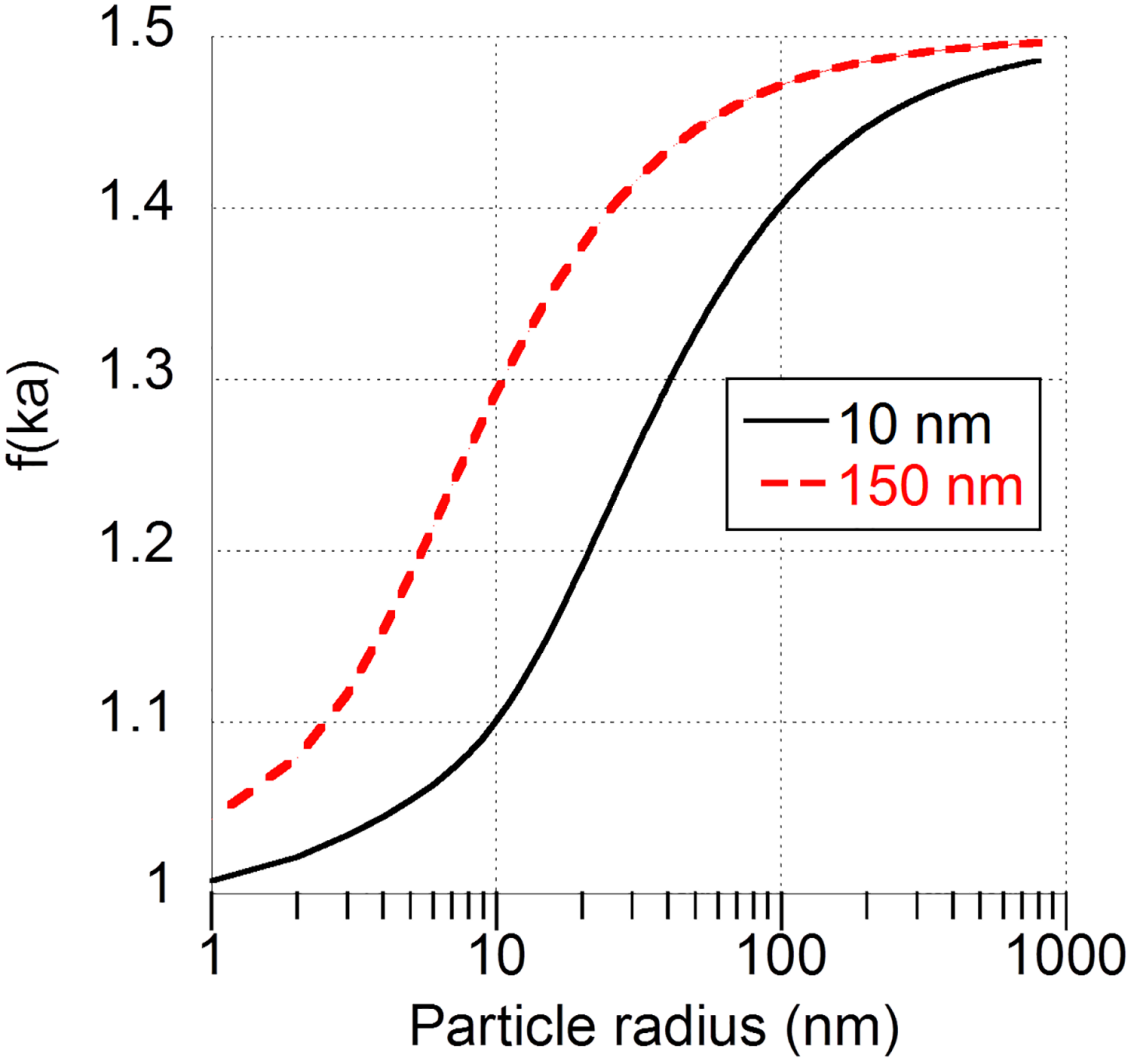
Plot illustrating the effect of particle/agglomerate size on Henry’s function f(κ·a) for NaCl solutions with 10 and 150 mM ionic strengths using the Ohshima correction (Eqs 3 and 4).

The ZP is influenced by the perturbation of the diffuse charge density due to particle motion, which in turn depends on the particle size [18]. A correction for this size dependence of the ZP can be estimated by using the Ohshima’s expression for the Henry function that takes into account the particle size (see section 3, Eqs 3 and 4) [7, 35]. This is illustrated in Fig 4, showing how much the increase in particle size changes the Henry function according to the Ohshima correction. Here, the change in Debye length (κ^-1^) as a function of ionic strength was also used in the calculations (Eq 1). Some examples for estimating the ZP from f(κ·a) in Fig 4, using a ZP of -18 mV in the Hückel approximation (f(κ·a) = 1), are given in Table 2. The ZPs presented in Fig 1 were in both cases calculated using the Smoluchowski approximation, *i*.*e*. both when the NP size remained at approximately 10 nm in the 10 mM NaCl solution and when a large fraction had agglomerated to a size of approx. 1000 nm in the 150 mM NaCl solution. The use of the Smoluchowski approximation was in that case merely selected to illustrate differences in the intensity curves for solutions of different ionic strength. As seen in Fig 4 and Table 2, the Smoluchowski approximation is relatively correct for the large (1000 nm) agglomerates in the 150 mM NaCl solution, while the Hückel approximation appears to be more suitable for the smaller (10 nm) non-agglomerated NPs in the 10 mM NaCl solution. Most correct would be to use the estimated f(κ·a), as calculated in Fig 4.

**Table 2:** Variations in f(κ·a) and zeta potential with changing particle size and ionic strength according to the Ohshima correction[7, 35] using values for Ag NPs in NaCl (Fig 3) compared with the Smoluschowski (κ·a>>1, f(κ·a) = 1.5) and the Hückel (κ·a<<1, f(κ·a) = 1) models. [35].

Model used	f(κ·a)	Zeta potential (mV)
**Smoluchowski**	1.5	-27.0
**Hückel**	1	-18.0
**Ohshima, 10 nm diameter (10 mM NaCl)**	1.05	-18.9
**Ohshima, 10 nm diameter (150 mM NaCl)**	1.25	-22.5
**Ohshima, 1000 nm diameter (10 mM NaCl)**	1.47	-26.5
**Ohshima, 1000 nm diameter (150 mM NaCl)**	1.49	-26.8

As seen in Table 2, the ZP varied from -22.5 to -26.8 mV for the different particle sizes in 150 mM NaCl (from Fig 4;10–1000 nm). This spread may contribute to the broadening of the ZP distribution curve displayed in Fig 2. However, it is not the only explanation since the broadening *e*.*g*. in Fig 2C is much larger (ca. 100 mV) compared to the size corrections (ca. 4 mV). Brownian motion can also contribute to the broadening of the zeta potential distribution [43]. As the Brownian broadening increases with decreasing particle size, the largest effects will be observed for nano-sized particles. A polydisperse distribution will thus also result in a broader zeta potential distribution as a result of Brownian motion broadening for differently sized NP agglomerates. Moreover, the signals from larger-sized agglomerates will be proportionally larger than from smaller agglomerates, conditions that further complicate the analysis of a polydisperse solution [18, 44]. In this case study, this means that the 1000 nm agglomerates are expected to predominantly govern the estimated ZP. These results are in line with previous investigations showing that highly polydisperse samples result in largely uncertain ZPs [20].

Besides the effects of agglomerate size on the perturbation of the diffuse charge density due to particle motion, there are also other reasons for differences in reported ZPs of particles of different size.[45] However, the effect of size is not apparent as there are counter-acting effects such as lower ZP for smaller particles at constant charge, and at the same time higher charge densities for smaller particles that result in higher ZPs.[45] In addition, agglomerates of NPs, as seen in *e*.*g*. Fig 3, may have different properties compared with mono-dispersed NPs, further complicating a stringent modelling of the ZP [46].

Measuring the zeta potential at different scattering angles could reduce the Brownian broadening [43, 47], and also help to deduce underlying reasons behind the broadening [48].

### Case study 2: Accurate ZP determination in the presence of biomolecules

**Fig 5:**
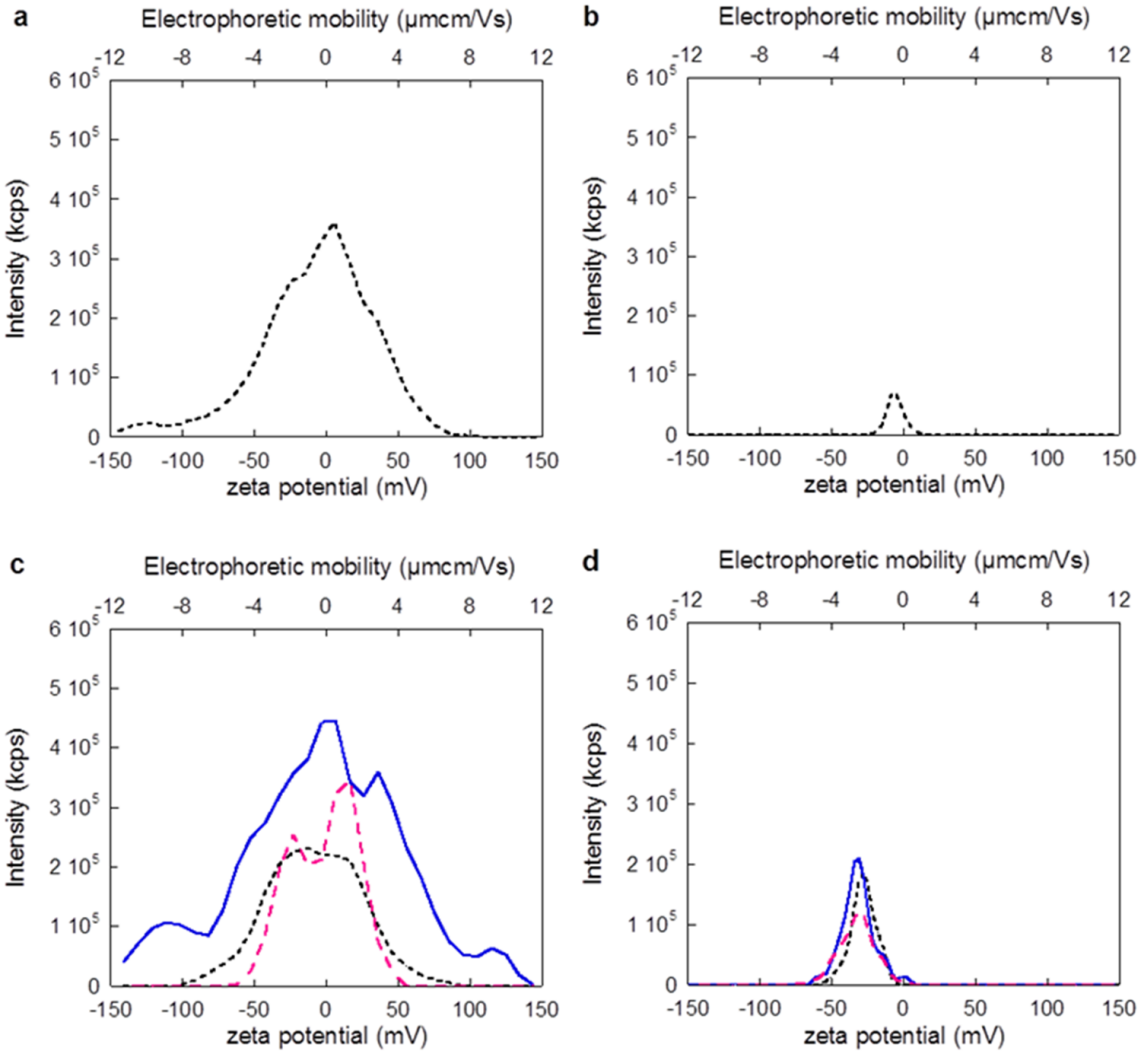
ZP intensity distributions in blank solutions without any particles: (a) cell media (BEGM) and (b) 10 mM NaCl, and corresponding intensity distributions in the same solutions with citrate-capped AgNPs: (c) cell media (BEGM) and (d) 10 mM NaCl. For the blank samples (a) and (b), average values of three independent measurements are shown, whereas corresponding three independent measurements are shown for the samples that contain particles, (c) and (d).

It can sometimes be difficult to distinguish the ZP of the NPs from the ZP of constituents of a biological fluid, such as proteins in cell media. The importance of using and analyzing blank samples in this context is hence crucial as demonstrated in this case study and illustrated in Fig 5 which displays intensity curves of ZP measurements conducted on citrate capped Ag NPs in cell media (BEGM) and in 10 mM NaCl compared with corresponding blank signals in each solution without any particles.

**Fig 6:**
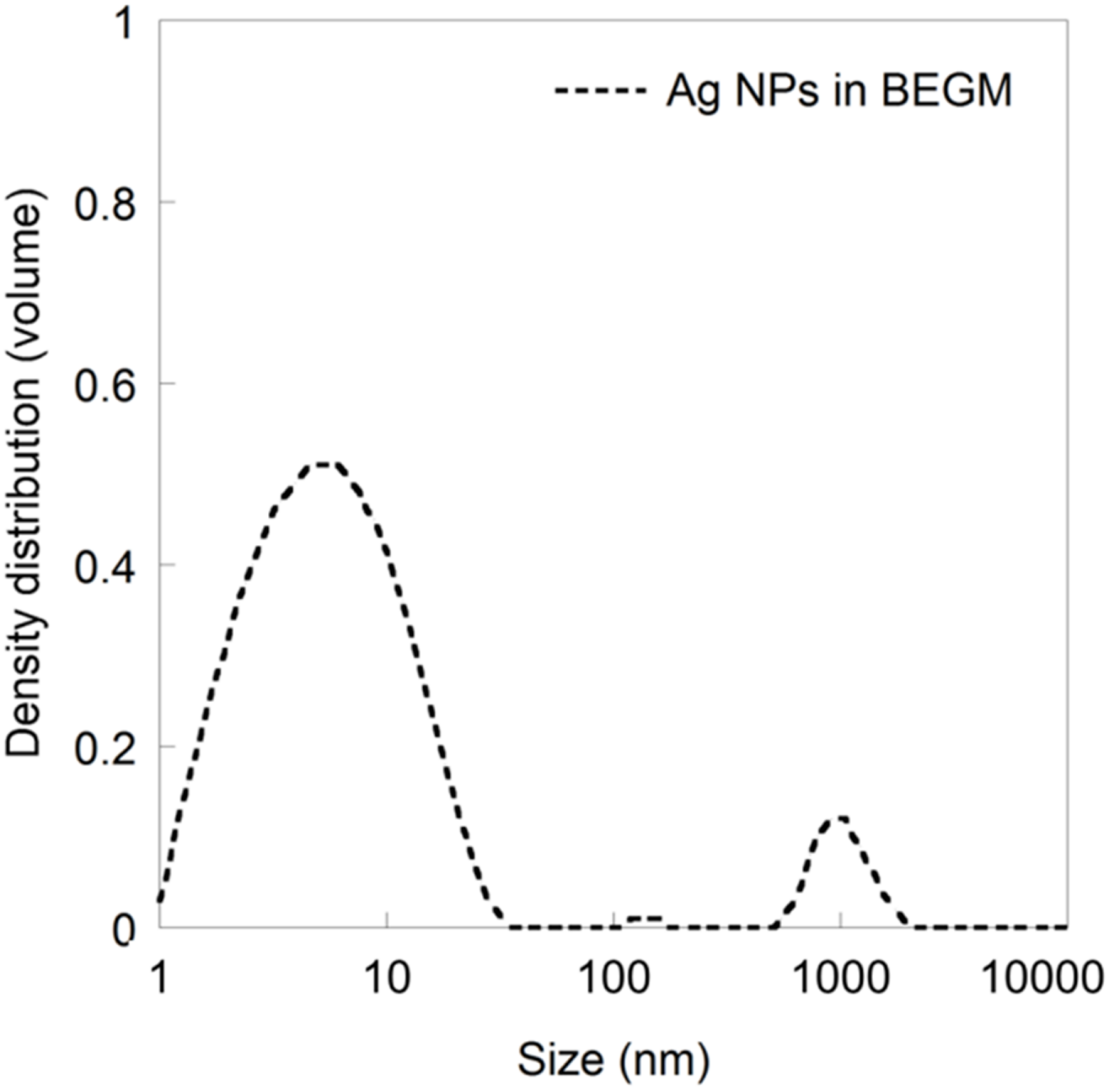
Particle size distribution (volume) of citrate-capped Ag NPs in BEGM cell media. Data from [24].

The blank samples of the cell medium BEGM show an intense, broad peak, whereas the blank samples in 10 mM NaCl barely show any signal at all. There is a clear shift in the ZP distribution peak when comparing the 10 mM NaCl blank with the 10 mM NaCl solution containing the Ag NPs. The BEGM medium contains proteins in the added cell growth factors that can contribute to the ZP signal.[49] The similar ZP observed with and without Ag NPs in BEGM renders hence that it is impossible to draw any reliable conclusions on such systems regarding the ZP. It is worth mentioning that measurements of the particle size distribution of the investigated particle concentration of Ag NPs (10 mgL^-1^) resulted in a high quality and well distinguishable signal for citrate-capped particles, as previously reported (Fig 6) [24].

In addition to the peak representing pristine 10 nm citrate-capped Ag NPs, the size distribution in Fig 6 shows agglomerates of a few 100 nm up to several 1000 nm, and thus a highly polydisperse suspension. As discussed above, the polydispersity complicates the determination of the ZP further and can contribute to the broadening of the ZP distribution curve. Most likely, constituents of the BEGM cell media interact with the Ag NPs, forming a surface layer of adsorbed biomolecules (bio-corona). This formation may result in a change in ZP either due to a charged, adsorbed layer, or a shift in the position of the slipping plane resulting from the adsorption of bulky molecules. The authors have previously speculated that the reasons for observed changes in the ZPs of Ag NPs when increasing the solution concentration of a bulky surfactant [50] to some extent are related to a shift in the position of the slipping plane. By combining the ZP measurements with information on the surface characteristics of the NPs (thickness, structure, composition) and the molecules present in solution, it may be possible to improve the interpretation of the ZP and deduce its origin [18]. Again, this highlights the importance to present ZP intensity distribution curves and not only average value since these curves provide information on the reliability of the data and acknowledge the problem of limitations for complex particle dispersions. Such data presentation is crucial for accurate interpretation and understanding of ZPs of NPs in for instance cell media used in nanotoxicological assessments.

### Case study 3: Effect of rapid particle agglomeration, sedimentation and dissolution on the zeta potential

**Fig 7:**
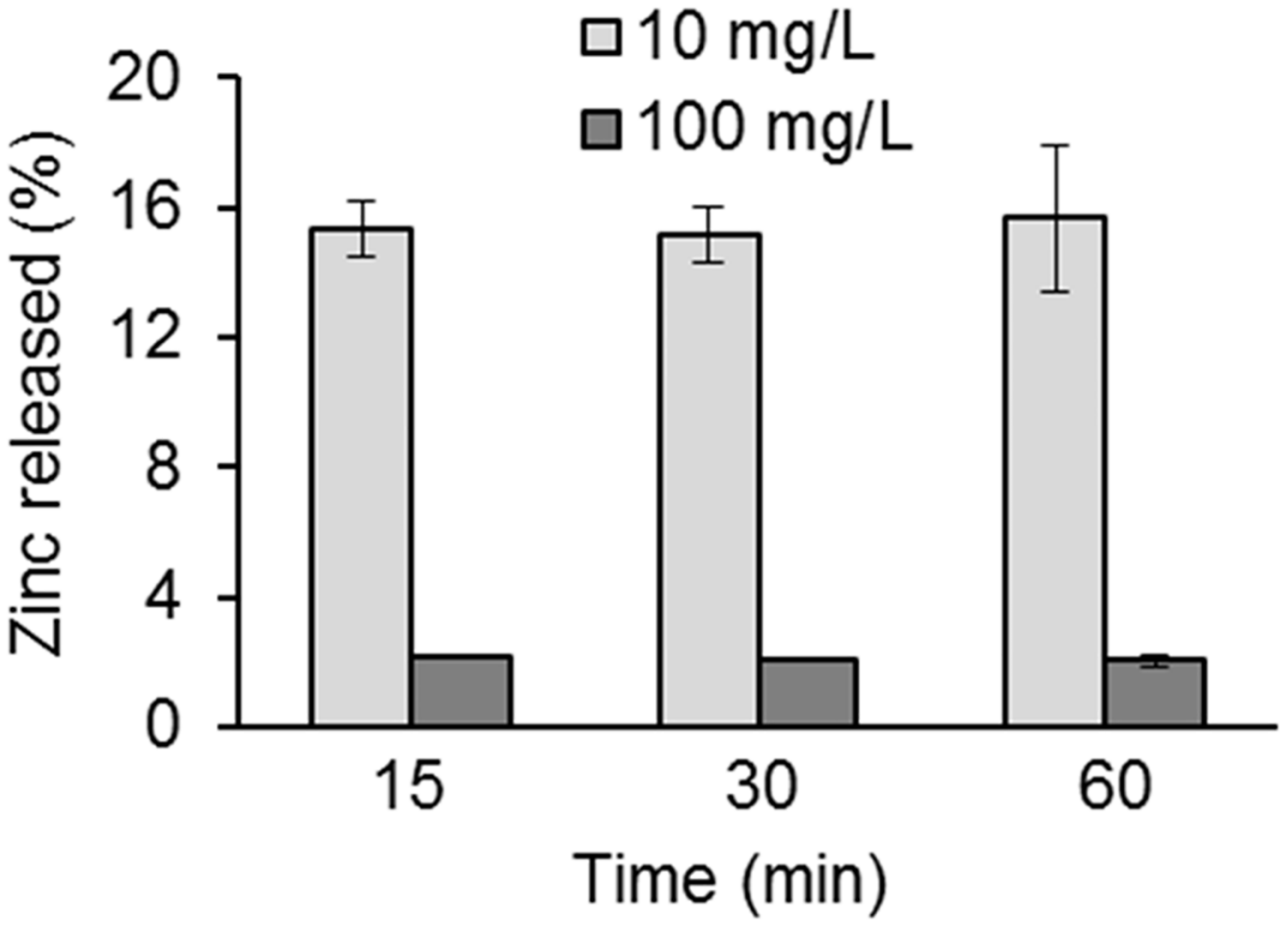
Fraction of released Zn, determined by means of AAS, compared to the total amount of Zn (added as Zn NPs: 10 mg L^-1^ and 100 mg L^-1^) in synthetic surface water after 15, 30 and 60 min of exposure.

This case study addresses difficulties to estimate the ZP of rapidly dissolving, agglomerating, and sedimenting NPs, in this case illustrated with Zn NPs in synthetic surface water. As seen in Fig 7, approximately 16 and 2% of the added Zn content (as NPs) were released into solution within 1 h for the 10 and 100 mgL^-1^ loadings, respectively. This corresponds to a concentration of dissolved Zn of approximately 1.6 and 2 mgL^-1^ for the 10 and 100 mgL^-1^ loadings, respectively and equivalent to an ionic strength of approximately 30 μM.

**Fig 8:**
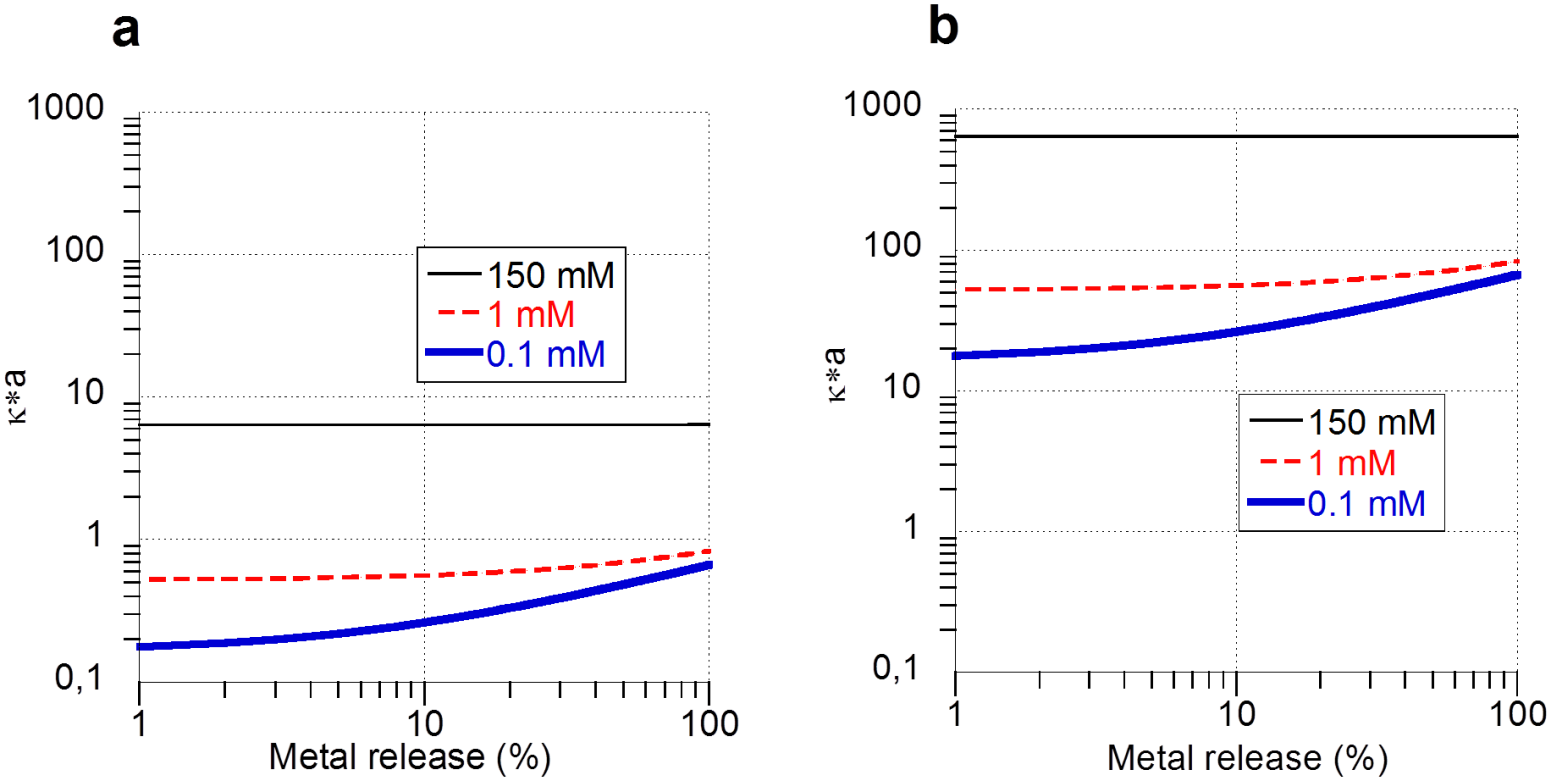
Simulation of changes in Henry’s function (κ·a), Eq 3, as a function of the fraction of metal release compared with the total amount of metal in the added Me NPs (0.1 gL^-1^) of two different particle sizes, a) Ø 10 nm and b) 1000 nm, in dispersion media of different ionic strength (0.1, 1, 150 mM).

In a low ionic strength solution, a high extent of metal dissolution can influence which model to use to accurately estimate the ZP, see section 3 for more details. The ionic strength of the solution increases with an increasing amount of metal dissolution, and result in an increased shielding of the electrostatics and a concomitant change in κ. This is demonstrated in Fig 8 that simulates the influence of ionic strength of rapidly dissolving Me NPs (loading 0.1 gL^-1^) on the κ·a parameter. The effect is most prominent at low ionic strengths, where the dissolving metal NP (1.5 mM total metal ions) significantly increases the ionic strength and shields the electrostatic interactions. In the solution of higher ionic strength (150 mM) on the other hand, the addition of metal ions does not have a strong effect on *κ*·*a* due to the inherent much larger ionic strength compared with the added ionic strength from dissolved metal NPs.

As shown in Fig 8, the Smoluchowski approximation (κ·a >>1) becomes more viable with increasing particle size and extent of metal dissolution.

**Fig 9:**
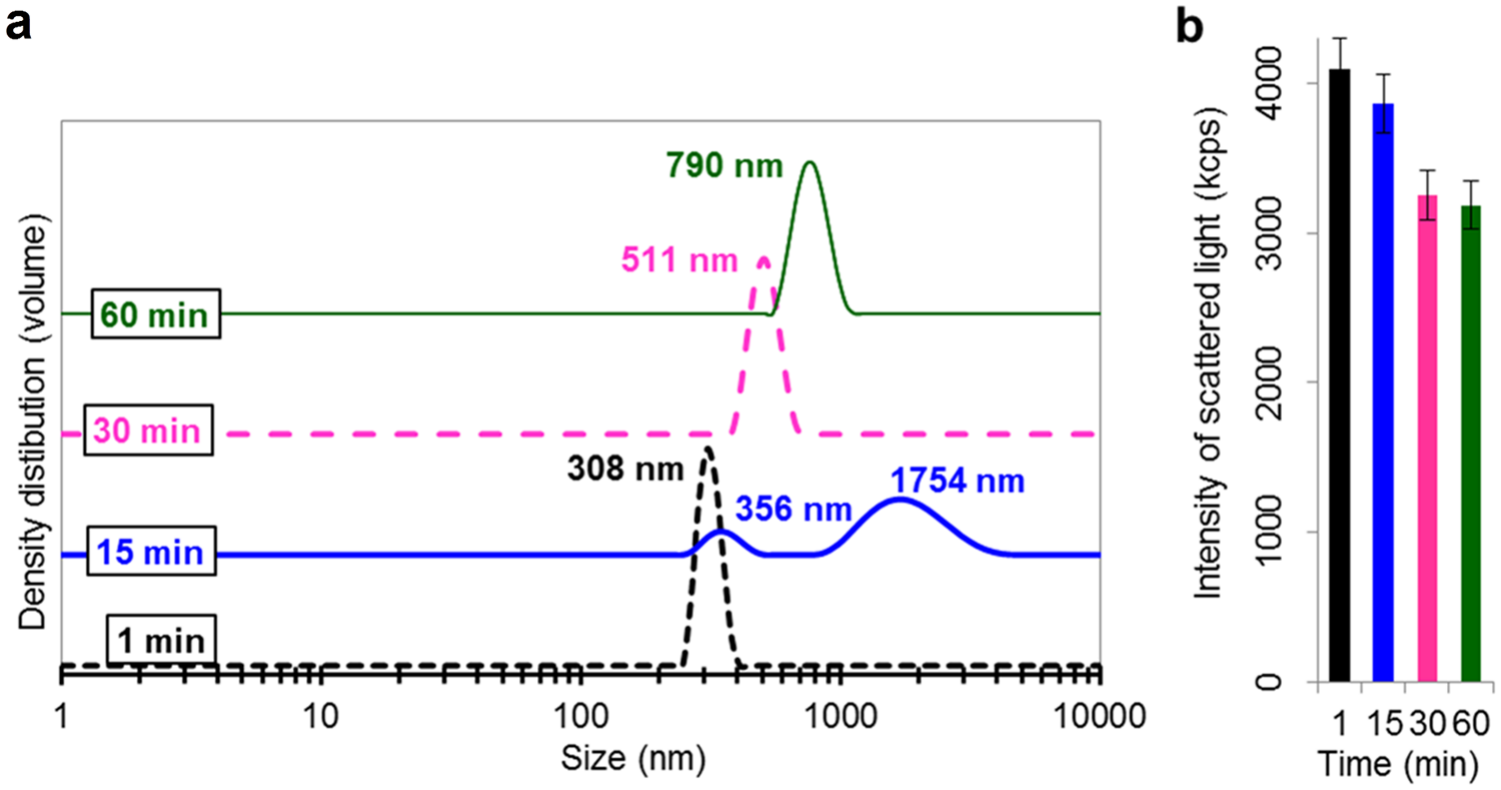
Particle size distribution (a) and scattered light intensity (b), as depicted by means of PCCS, of Zn NPs in synthetic surface water (10 mgL^-1^) after 1, 15, 30 and 60 min of exposure.

In the case of the Zn NPs in synthetic surface water with particle agglomerate sizes of several hundred nm (Fig 9) and a low ionic strength (approx. 2 mM), the dissolution (approx. 2% after 1 h at a 100 mgL^-1^ loading, and 16% after 1 h at a 10 mgL^-1^ loading) did not have any significant effect on the choice of the ZP modelling results. This is seen from the fact that κ·a was still >>1 after the addition of approx. 0.02 mM Zn ions (approx. 2% for the 100 mgL^-1^ particle loading), as seen in Fig 8b, making the Smoluchowski approximation still valid.

In general, it can be concluded that the choice of model for ZP predictions is crucial for conditions with high loadings of metal NPs (*e*.*g*. 0.1 g/L, or approx. 1.5 mM) in solutions of low ionic strengths. These conditions would result in a situation similar to the 0.1 mM ionic strength curves illustrated in Fig 8. Another option for these conditions would be to use the Ohshima correction (Eq 2) for particle size and directly obtain a number for the Henry function.

In media of high ionic strength, *e*.*g*. cell media used in nanotoxicological assays that have an ionic strength of approx. 150 mM, the effect of increased amounts of metals in solution will not largely influence the κ·a parameter and hence not the choice of model for the ZP calculation. This effect is seen in Fig 8 from the flat curve of the solution of high ionic strength (150 mM).

Sedimentation of the largest particles with time can cause that reduced particle sizes are measured with time. At the same time, particle agglomeration can result in measurements of increased particle sizes. This is illustrated in Fig 9 that shows a reduction of the scattered light intensity with time despite the formation of larger particle agglomerates with time. Both changes are statistically significant when comparing the 1 and 60 min exposure time (p<0.05, Student’s t-test). Conditions without any sedimentation would result in an increased intensity of the scattered light since it is proportional to 10^6^ of the particle size. After 15 min, Fig 9, the particles have formed an additional size distribution consisting of large agglomerates sized approximately 1800 nm. Continued sedimentation was observed beyond the 15 min time point. These changes in agglomeration with time may affect the choice of model for the determination of the ZP (see *e*.*g*. Fig 4). In addition, particle sedimentation reduces the concentration of NPs in solution and may decrease the signal-to noise ratio.

### Case study 4: Influence of sonication of NP dispersions on the zeta potential

This case illustrates the influence of sonication, a common method (*e*.*g*. probe, ultrasonic bath) to disperse NPs, on the measured ZP. Sonication has previously been shown to highly influence NP characteristics, in particular for bare metallic particles susceptible to oxidation [51, 52]. There is however no clear general trend in neither the change in magnitude of ZP upon sonication nor the change of ZP in the positive or negative direction, as the influence is highly material specific [20, 53]. There are also studies in which a prolonged sonication (increased delivered acoustic energy) did not influence the ZP further compared to initial effects.[27, 54] The situation is clearly complex, as sonication can influence the ZP in several ways. Some examples are given in the following on conditions for which sonication can influence the ZP:

- The reduction in particle size as a result of metal dissolution (or disintegration of agglomerates) induced by the sonication process [28, 55, 56]. As previously discussed, a change in particle size can change the ZP, depending on prevailing conditions.
- The sonication step may influence the shape and morphology of the NPs [56, 57]. As the common models assume spherical particles, a substantial change in *e*.*g*. shape can influence the ZP.
- Surface oxide properties can be changed due to sonication [56, 57]. This will then also influence the ZP as different surface oxides *e*.*g*. can have different surface pKa:s and hence different isoelectric points (IEP)[58] that will shift the ZP.

As an example, Cu NPs are in the following used to elucidate the effect of sonication on the ZP. The ZP of Cu NPs has previously been seen to be shifted in the positive direction upon sonication [53]. Table 3 shows XPS data of the same batch of unexposed Cu NPs and non-sonicated and sonicated Cu NPs in ultrapure water for 15 min.

**Table 3:** XPS data on Cu 2p and O 1s for dry, sonicated, and non-sonicated Cu NPs.

Cu NPs	Binding energy Cu 2p (eV)	Assignment	Binding energy O 1s (eV)	Assignment [59]	OH^-^/O^2-^, mass ratio	Particle size in Ultra-pure water (nm)
**dry powder**	933.3±0.2	Cu(II)	529.2±0.3	OH^-^	1.1±0.5	^a^
			530.6±0.4	O^2-^		
**non-sonicated**	931.6±0.2	Cu(0,I)	529.5±0.3	OH^-^	1.5±0.6	800±51
	933.3±0.3	Cu(II)	531.0±0.5	O^2-^		
**sonicated**	933.2±0.2	Cu(II)	529.2±0.3	OH^-^	2.8±0.3	300±62
			531.1±0.4	O^2-^		

^a^ no results obtained due to the formation of large agglomerates and sedimentation.

The results show that the OH^-^/O^2-^ ratio increases upon sonication (p<0.05, Student’s t-test), as deduced from comparing the non-sonicated Cu NPs exposed in water and the dry unexposed Cu NPs with Cu NPs sonicated in water. The results imply a change in the surface oxide, composition with an oxide more rich in OH-groups after sonication. This is also seen from the Cu 2p peak with Cu(II) predominating the outermost surface (5–10 nm) of the sonicated particles compared with signals attributed to both Cu(0,I) and Cu(II) on the non-sonicated particles immersed in water. Sonication hence seems to result in an increased surface oxide thickness (lack of Cu(0,I) signal). As Cu(OH)_2_ have been seen to have a higher IEP compared with CuO (present on the Cu NP surface)[58], this increase in OH^-^/O^2-^ can, assuming all else equal, shift the ZP in the more positive direction. However, as seen previously [28, 55], sonication in general reduces the size of particle agglomerates. The effect of particle size on the ZP, discussed above, is hence entangled with the effect on the surface oxide. The sizes of the sonicated Cu NPs were 300 nm and the non-sonicated were 800 nm. The Ohshima correction for ZP due to different particle sizes, as estimated from Fig 4 and Eqs 3 and 4, only gave a very minor difference between the sonicated and non-sonicated Cu NPs (approx. 5% difference).

### Concluding remarks

The aim of this paper has been to elucidate how measurements of the zeta potential of metal NPs in solutions of different characteristics are influenced by the physico-chemical properties of the solution, the particle characteristics and the experimental conditions. General guidelines on how to improve the knowledge related to such measurements and how generated data should be interpreted and reported are compiled below:

- A single value (the mean value) of the zeta potential is often reported without any information given about the distribution of the potentials. The mean value does not give the complete picture and does not provide enough information on the quality of the data. A narrow intensity distribution curve that does not differ too much between the measurements and that shows minor noise indicates reliable and consistent data, whereas a measurement is less reliable when there is a broad distribution and a large deviation in appearance between replicate measurements. In addition to mean values, reporting of zeta potentials should hence also include intensity distribution curves that justify the quality and reliability of the measurements. Intensity distribution curves for the blank solution without particles should further be reported.
- The electrophoretic mobility should be presented in addition to the zeta potential in order for the reader to calculate the zeta potential using another choice of method for the calculation.
- Details regarding the sonication procedure should be reported, preferably in a similar way as recommended by Taurozzi *et al*.[51] This will enable repeatability of experiments, which is important since the sonication process can influence the zeta potential.
- In order to improve the interpretation of the zeta potential, additional information on the extent of metal release, the size distribution and agglomeration behavior of the metal NPs, presence of coatings/capping agents, ionic strength, and biomolecules present in solution should be documented and reported. The choice of model for calculating the zeta potential should be carefully made based on this information.

## Supporting information

S1 Fig XPS spectra of Cu 2p for Cu NPs of the different investigated exposure and preparation: Unexposed, exposed in ultrapure water for 15 min, sonicated in ultrapure water for 15 min. (DOCX)
