# Supplementary material for: Difficulties and flaws in performing accurate determinations of zeta potentials of metal nanoparticles in complex solutions—Four case studies
Source: PLoS One. 2017 Jul 27;12(7):e0181735. doi: 10.1371/journal.pone.0181735 (PMC5531457; doi:10.1371/journal.pone.0181735)
Supplement: S1 Fig — (DOCX) [file pone.0181735.s001.docx]

**Supporting information**

# Difficulties and flaws in performing accurate determinations of zeta potentials of metal nanoparticles in complex solutions – Four case studies

*Sara Skoglund,^1^ Jonas Hedberg,^1^* Elena Yunda,^1,2^* [*Anna Godymchuk*](https://www.researchgate.net/profile/A_Godymchuk)*,^2,3^ Eva Blomberg,^1,4^ Inger Odnevall Wallinder^1^*

^1^KTH Royal Institute of Technology, Division of Surface and Corrosion Science, School of Chemical Science and Engineering, SE-100 44 Stockholm, Sweden

^2^National Research Tomsk Polytechnic University, Tomsk 634050, Russia

^3^National University of Science and Technology “MISIS”, Moscow 119991, Russia

^4^RISE Research Institutes of Sweden, Chemistry, Materials and Surfaces, Sweden

*Corresponding author:

e-mail: [jhed@kth.se](mailto:jhed@kth.se) (JH)

**X-ray photoelectron spectroscopy**

Figure S1. XPS spectra of Cu 2p for Cu NPs of the different investigated exposure and preparation: unexposed, exposed in ultrapure water for 15 min, sonicated in ultrapure water for 15 min.
